# Lateral homogeneity of the electronic properties in pristine and ion-irradiated graphene probed by scanning capacitance spectroscopy

**DOI:** 10.1186/1556-276X-6-109

**Published:** 2011-01-31

**Authors:** Filippo Giannazzo, Sushant Sonde, Emanuele Rimini, Vito Raineri

**Affiliations:** 1 CNR-IMM, Strada VIII, 5, Zona Industriale, 95121, Catania, Italy; 2 Scuola Superiore di Catania, Via San Nullo, 5/I, 95123, Catania, Italy; 3 Department of Physics and Astronomy, University of Catania, Via S. Sofia, 95123, Catania, Italy

## Abstract

In this article, a scanning probe method based on nanoscale capacitance measurements was used to investigate the lateral homogeneity of the electron mean free path both in pristine and ion-irradiated graphene. The local variations in the electronic transport properties were explained taking into account the scattering of electrons by charged impurities and point defects (vacancies). Electron mean free path is mainly limited by charged impurities in unirradiated graphene, whereas an important role is played by lattice vacancies after irradiation. The local density of the charged impurities and vacancies were determined for different irradiated ion fluences.

## Introduction

Graphene, a two-dimensional (2D) sheet of carbon atoms in a honeycomb lattice, attracted the interest of the nanoelectronics scientific community for its remarkable carrier transport properties [1,2]. Ideally, in a free-standing graphene sheet without lattice defects and adsorbed impurities, charge carriers can exhibit a giant intrinsic mobility [2] and can travel for micrometers without scattering at room temperature. As a matter of fact, very high values of mobility (>2 × 10^5 ^cm^2 ^V^-1^s^-1^) and electron mean free path have been observed only in vacuum and at low temperature (5 K) in "suspended" graphene sheets obtained by mechanical exfoliation of highly oriented pyrolytic graphite (HOPG) [3]. The mobility values measured at room temperature commonly reported in the literature range from approximately 2 to 2 × 10^4 ^cm^2 ^V^-1^s^-1^, depending on the graphene synthesis methods [1,4], on the kind of substrate on which it is deposited [5], and on the processing conditions used to fabricate the test patterns for electrical characterization. This large variability is a clear indication that the intrinsically outstanding transport properties of graphene are severely limited by extrinsic factors, like the presence of charged impurities, lattice defects and, more generally, by lattice disorder (including local strain). Single layers of graphene (SLG) obtained by mechanical exfoliation of HOPG [1] typically exhibit a very high crystalline order, whereas a high-defect density is present both in epitaxial graphene growth by thermal decomposition of SiC [6] and in graphene obtained by chemical reduction of graphene oxide [7].

Recently, the intentional production of defects in selected areas of a graphene sheet has also been proposed as a method to locally modulate the transport properties. Several methods, like plasma treatments [8], and electron [9] or ion irradiation [10], have been used for this aim. Recently, it has been reported that graphene hydrogenation by exposure to atomic hydrogen resulted in the conversion of graphene, a zero bandgap semiconductor, to graphane, a two-dimensional insulator [11]. Among all these methods, ion irradiation allows a better control through a precise definition on the ion energy and fluence. Spectroscopic characterization methods, like micro Raman spectroscopy (μR), are the commonly used techniques to evaluate the density of defects in a graphene sheet. The characteristic D line at 1360 cm^-1 ^in the Raman spectra is a fingerprint of defects/disorder in the crystalline lattice of graphitic materials. However, the lateral resolution of μR is limited by the laser spot size (typically in the order of 0.5-1 μm). In this article, we present a scanning probe method based on nanoscale capacitance measurements to determine locally (on 10-100 nm scale) the electron mean free path in pristine and in ion-irradiated graphene with different ion fluences. The impurity and vacancy densities on the probed area were extracted by fitting the experimental results with models of electron scattering by Coulomb impurities and lattice defects.

## Experimental details

Graphene samples obtained by mechanical exfoliation of HOPG were deposited on a n^+^-Si substrate covered with 100 nm SiO_2 _[12]. Optical microscopy, tapping mode atomic force microscopy (AFM) and μR spectroscopy were used to identify SLG [13]. Some of the as-deposited (pristine) samples were then irradiated with C^+ ^ions at 500 keV. Irradiations of the samples with C^+ ^ions were carried out under high vacuum conditions (10^-6 ^Torr) to minimize surface contaminations. At 500 keV energy, the projected range of the C^+ ^ions is approximately 1 μm, quite deep into the n^+^-Si substrate. This minimizes the damage both in the 100 nm SiO_2 _layer and at the interface between SiO_2 _and n^+ ^Si. Infact, a quality of SiO_2 _and SiO_2_/Si interface comparable to that of non-irradiated samples is crucial for the capacitance measurements discussed later. Different C^+ ^ion fluences, ranging from 1 × 10^13 ^to 1 × 10^14 ^ions/cm^2^, were used for irradiation [14].

The lateral homogeneity of the electronic transport properties both in pristine and ion-irradiated graphene was investigated by local capacitance measurements on the graphene/SiO_2_/n^+^Si stack, using scanning capacitance spectroscopy (SCS) [12,15].

**Figure 1:**
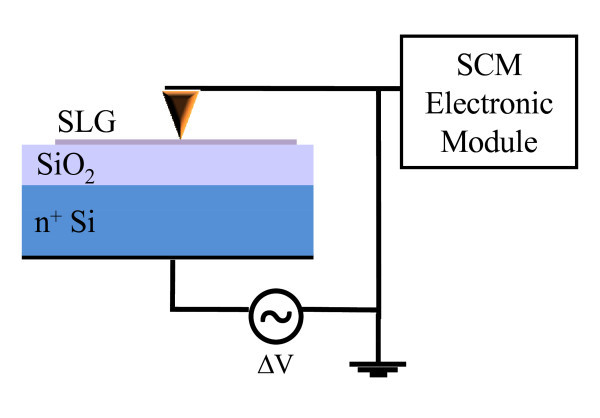
**Schematic representation of the scanning capacitance spectroscopy setup**.

Scanning capacitance spectroscopy (SCS) was performed at room temperature using a DI3100 AFM by Veeco equipped with Nanoscope V electronics and with the scanning capacitance microscopy (SCM) head. SCS is an extension of the conventional SCM [16-19]. In SCS, the conductive AFM tip is placed on a discrete array of positions, lifting the tip by 20 nm at every interval. This "step and measure" approach eliminates the lateral (shear) force usually present when tip is scanned on a surface. Moreover, the vertical contact force can be suitably minimized to get a good electrical contact to the graphene layers while avoiding damage at the same time. A modulating bias Δ*V *= *V*_g_/2(1 + sin(*ωt*)), with amplitude *V*_g _in the range from -1.2 to 1.2 V and frequency *ω *= 100 kHz, was applied between the Si n^+ ^backgate and the nanometric contact on graphene represented by a Pt-coated Si tip (see schematic in Figure 1). The ultra-high-sensitive (10^-21 ^F/Hz^1/2^) capacitance sensor connected to the conductive AFM tip measures, through a lock-in system, the capacitance variation Δ*C *induced by the modulating bias.

## Results and discussion

**Figure 2:**
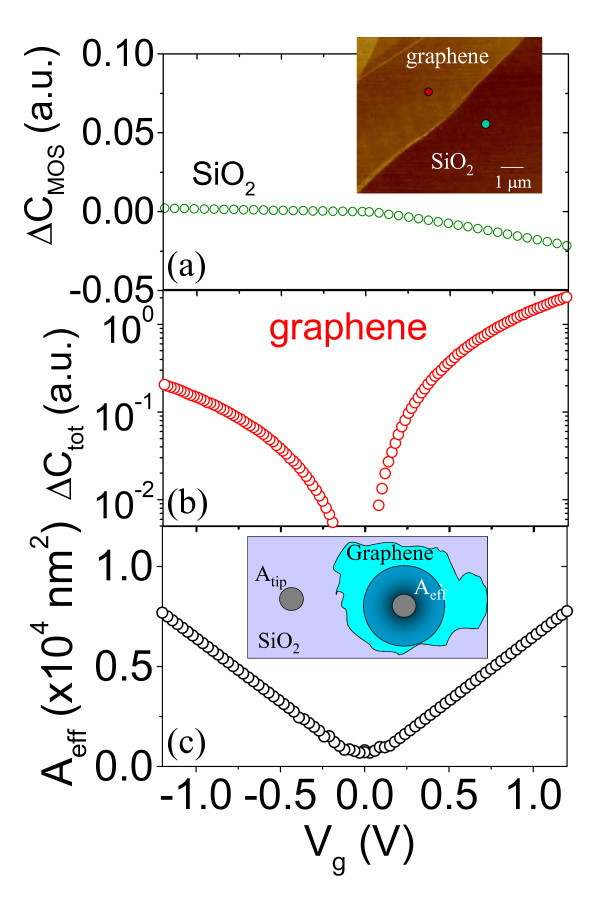
**Evaluation of the effective area from local capacitance measurements**. Local capacitance-voltage curves measured on fixed positions on bare SiO_2 _**(a) **and on graphene-coated SiO_2 _**(b) **for a sample not subjected to ion irradiation. AFM morphology of a graphene flake on SiO_2_, with indicated the probed positions by the SCS tip. (inset of a). Effective area evaluated from the *C*-*V *curves in (a) and (b). Schematic representation of *A*_tip _and *A*_eff _(inset of c).

In Figure 2, capacitance-voltage curves measured on fixed positions on bare SiO_2 _and on graphene-coated SiO_2 _are reported for a sample not subjected to ion irradiation. The tip positions are indicated in the AFM image in the inset of Figure 2a. When the tip is in contact on bare SiO_2_, a typical capacitance-voltage curve for a metal-oxide-semiconductor (MOS) capacitor from accumulation (at negative sample bias) to depletion (at positive sample bias) is measured (see Figure 2a). The area of the MOS capacitor is represented by the tip contact area *A*_tip_, as illustrated in the insert of Figure 2c. When tip is in contact on graphene, the measured capacitance is minimum around zero bias and increases both for negative and positive bias (see Figure 2b). At *V*_g _= 0, the Fermi level in graphene is almost coincident with the Dirac point. A positive modulating bias between the substrate and the tip locally induces a shift of the graphene quasi-Fermi energy *E*_F _in the conduction band, and, hence, an accumulation of electrons at the nanometric tip/graphene contact. On the contrary, a negative bias induces a shift of *E*_F _in the valence band, and, hence, an accumulation of holes at the tip/graphene contact. The carrier density n induced by the gate bias *V*_g _can be expressed as *n *= *C*_ox_'*V*_g_/*q*, where *q *is the electron charge, and *C*_ox_' is the oxide capacitance per unit area (*C*_ox_' = *ε*_ox_*ε*_0_/*t*_ox_, being *ε*_0 _the vacuum permittivity, *ε*_ox _= 3.9 and *t*_ox _are the relative permittivity and the thickness of the SiO_2 _film, respectively). The value of *E*_F _can be related to the applied bias as *E*_F _= *ħv*_F_*k*_F_, being *k*_F _= (πn)^1/2^, *ħ *the reduced Planck's constant, and *v*_F _= 1 × 10^6 ^m/s, the electron Fermi velocity in graphene. The induced charge n spreads over an area, *A*_eff_, which can be thought as the tip-graphene-insulator-semiconductor capacitor effective area (as schematically illustrated in the insert of Figure 2c). The effective area *A*_eff _can be evaluated from the ratio of the capacitance measured with the probe on graphene-coated regions (|Δ*C*_gr_|) and on bare SiO_2 _regions (|Δ*C*_ox_|) [15], i.e., *A*_eff _= *A*_tip_|Δ*C*_gr_|/|Δ*C*_ox_|, where the tip contact area *A*_tip _can be independently determined by scanning electron microscopy (*A*_tip _= 80 nm^2 ^in the present case). The evaluated *A*_eff _is reported as a function of the gate bias in Figure 2c. Except for *V*_g _= 0, *A*_eff _increases linearly with |*V*_g_| both for negative and positive *V*_g _values.

**Figure 3:**
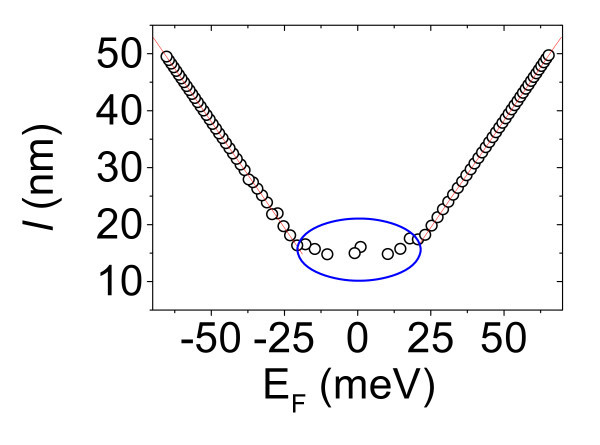
**Local electron mean free path versus the Fermi energy in a selected position on pristine graphene**.

It has been recently demonstrated that the effective area *A*_eff _obtained by local capacitance measurements is related to the local electron mean free path *l *in graphene by *A*_eff _= π*l*^2 ^[20]. In Figure 3, *l *is reported versus the evaluated Fermi energy. It can be noted that *l *is almost independent of *E*_F _close to the Dirac point. The behavior close to the Dirac point is consistent with the common adopted picture of the 2DEG split in a landscape of adjacent "electron-hole puddles" [21]. Close to the Dirac point, the effect of a gate bias is limited to a redistribution of carriers between the electrons and holes puddles without significantly changing the total carrier density. Figure 3 shows also that, for |*E*_F_| > 25 meV, *l *increases linearly with *E*_F _both in the hole and electron branches. This linear dependence gives indication on the main scattering mechanisms limiting *l *in our graphene samples.

Recently, expressions of the energy dependence of *l *have been determined for the different scattering mechanisms in the framework of a semiclassical model based on the Boltzmann transport theory [22]. The electron mean free path limited by scattering with graphene acoustic phonons (*l*_phon_) can be expressed as [22]

(1) $$l_{\text{phon}}\left(E_{\text{F}}\right)=\frac{\hbar^{3}\rho v_{\text{s}}^{2}v_{\text{F}}^{3}}{D_{\text{A}}^{2}kT}\frac{1}{E_{\text{F}}}$$

where *ρ *is the graphene density (*ρ *= 7.6 × 10^-^^7 ^kg/m^2^) [2], *D*_A _is the acoustic deformation potential (*D*_A _= 18 eV) [2], *v*_s _is the sound velocity in graphene [2], *k*_B _is the Boltzmann constant, and *T *is the absolute temperature.

The electron mean free path limited by Coulomb scattering with charged impurities (*l*_ci_) can be expressed as [22]

(2) lci(EF)=16ε02ε2ℏvFZ2q4Nci(1+q2πℏvεF0ε)2EF.

where *ε *= 2.4 is the average between *ε*_ox _and the vacuum relative dielectric constant, *Z *is the net charge of the impurity (it will be assumed *Z *= 1), and *N*_ci _is the density of impurities.

Finally, the electron mean free path for scattering by vacancies (*l*_vac_) can be expressed as [22]

(3) $$l_{\text{vac}}\left(E_{\text{F}}\right)=\frac{E_{\text{F}}}{\pi^{2}\hbar N_{\text{vac}}v_{\text{F}}}{\left[\ln\left(\frac{E_{\text{F}}}{\hbar v_{\text{F}}}R_{0}\right)\right]}^{2}$$

where *N*_vac _is the density of vacancies in graphene and *R*_0 _is the vacancy radius, that we assumed to be coincident with the *C*-*C *distance in the graphene plane (approximately 0.14 nm).

The experimentally determined linear dependence of *l *on *E*_F_, far from the Dirac point, suggests that scattering with charged impurities and/or point defects, e.g., vacancies, can be assumed as the main mechanisms limiting electron mean free path.

In this pristine graphene sample, the density of defects is negligible, as confirmed by the absence of the characteristic *D *peak in micro-Raman spectra. Hence, charged impurities, either adsorbed on graphene surface, or located at the interface with SiO_2 _substrate, can be assumed as the main scattering source liming *l*. The density of charged impurities in the probed position can be estimated by fitting the experimental curves in Figure 3 with Equation 2. The best fit (red line) is obtained with *N*_ci _= 49 × 10^10 ^cm^-2 ^both for the holes and the electron branch.

**Figure 4:**
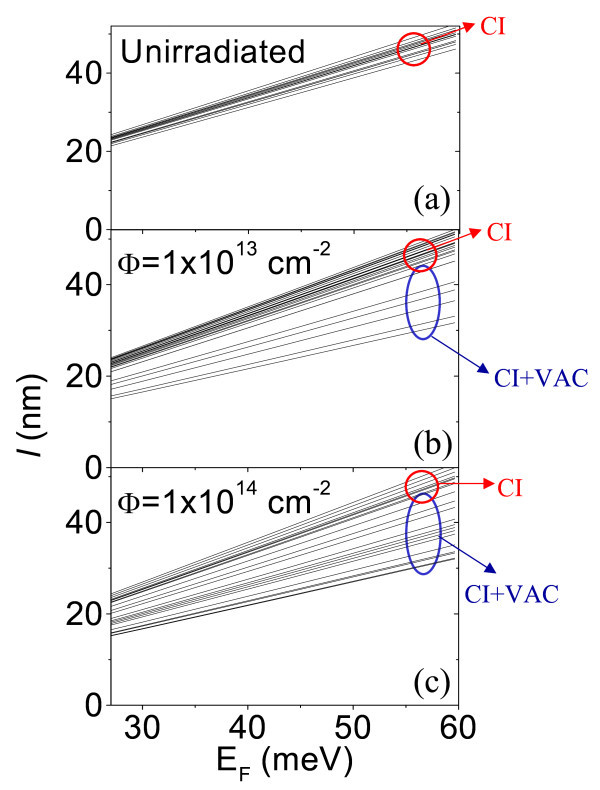
**Local electron mean free path versus the Fermi energy measured on array of several tip positions on pristine and irradiated graphene at different fluences**. On pristine graphene **(a)**. On irradiated graphene with 500 keV C^+ ^ions at fluences 1 × 10^13 ^cm^-2 ^**(b) **and 1 × 10^14 ^cm^-2 ^**(c)**, respectively.

**Figure 5:**
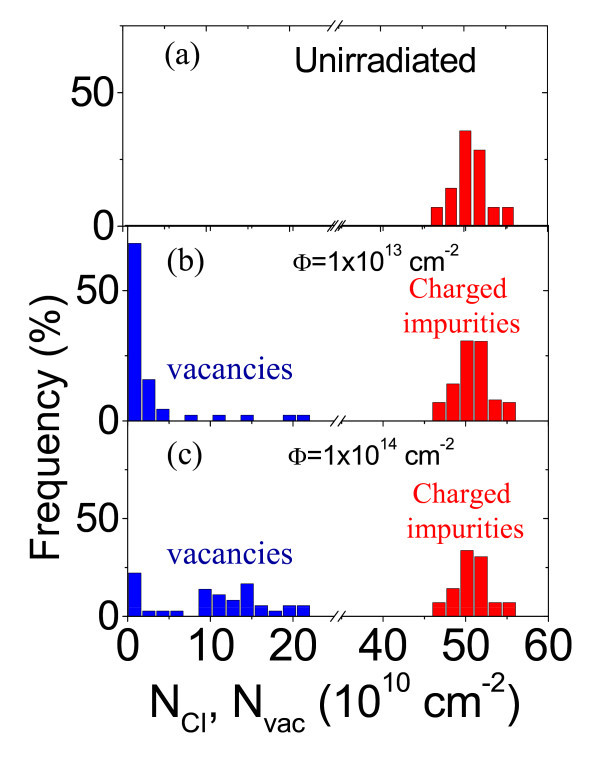
**Histograms of the locally measured densities of charged impurities and vacancies in pristine and ion irradiated graphene**. Charged impurities density in pristine graphene **(a)**. Charged impurities and vacancy densities in irradiated graphene with 500 keV C^+ ^ions at fluences 1 × 10^13 ^cm^-2 ^**(b) **and 1 × 10^14 ^cm^-2 ^**(c)**, respectively.

In Figure 4a, *l *versus *E*_F _measured on an array of 5 × 5 tip positions on pristine graphene is reported. By fitting each curve of the array with Equation 2, the local density *N*_ci _for each probed position can be extracted. The histogram of the charged impurity density on the analyzed area is reported in Figure 5a. It exhibits a Gaussian distribution peaked at 〈*N*_ci_〉 = 50 × 10^10 ^cm^-2 ^and with FWHM of 4 × 10^10 ^cm^-2^.

In Figure 4b,c, the measured *l *versus *E*_F _is reported for two arrays of tip positions on graphene samples irradiated with two different ion fluences, i.e., *Φ *= 1 × 10^13 ^cm^-2 ^and *Φ *= 1 × 10^14 ^cm^-2^. Comparing the set of curves in Figure 4a, i.e., for pristine sample, with those on Figure 4b,c, it is evident that the lateral inhomogeneity in the *l *values increases with the irradiated fluence. However, it is worth noting that two groups of *l*-*E*_F _curves can be distinguished for irradiated samples: (i) a first group, with *l *values comparable to those in the pristine sample, (ii) a second group with reduced mean free path. We assumed that *C *irradiation causes the formation of point defects (vacancies), whereas the density of charged impurities adsorbed on the graphene surface or at the interface with the substrate remains almost unchanged. Hence, the first group of curves in Figure 4b,c can be associated to the probed positions on the graphene surface without or with a very low density of point defects, whereas the second group associated to the probed positions with point defects. For the first group of curves, *l *can be fitted using Equation 2. The histograms of the *N*_ci _values determined in the probed positions is reported in Figure 4b,c, red bars, for the lowest and highest doses, respectively. It is worth noting that the *N*_ci _distributions in irradiated samples are very similar to those of non-irradiated sample. For the second group of curves in Figure 4b,c, *l *is limited both by charged impurities and vacancies scattering, i.e.,

(4) $$l^{-\text{1}}=l_{\text{ci}}{}^{-\text{1}}+l_{\text{vac}}{}^{-\text{1}}$$

For simplicity, an average value of the charged impurities density will be assumed in those positions (〈*N*_ci_〉 = 50 × 10^10 ^cm^-2^), and the local vacancy density was determined from Equations 2-4 using *N*_vac _as the fitting parameter. The distributions of the vacancy densities in the probed positions are reported in Figure 5b,c, blue bar, for the two fluences. It is worth noting, that, while in graphene irradiated with the lowest fluence *N*_vac _is higher than 2.5 × 10^10 ^cm^-2 ^(i.e. more than one vacancy on the probed area at *V*_g _= 1*V*) on only 16% of the probed positions, in graphene irradiated with the highest fluence *N*_vac _> 2.5 × 10^10 ^cm^-2 ^on more than 75% of the probed positions.

**Figure 6:**
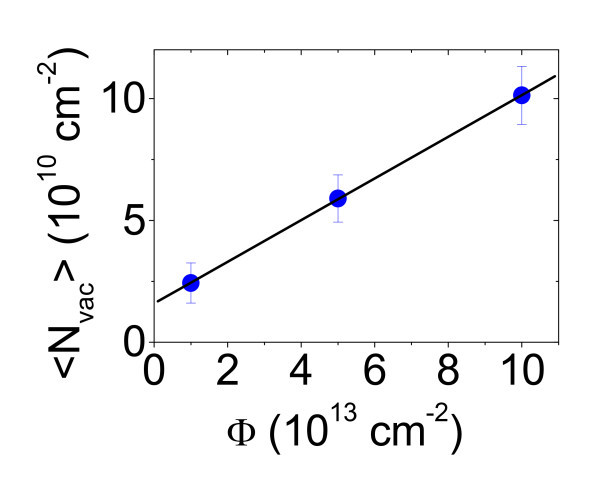
**Average vacancy density as a function of the irradiated fluence**.

For each fluence, the weighted average of the vacancy density on the probed area can be obtained by $\langle N_{\text{vac}}\rangle =\sum_{i=1}^{n}N_{\text{vac},i}f_{i}$, being *N*_vac,__*i *_the values of the vacancy densities in the histograms and *f*_*i *_the associated frequencies. The obtained 〈*N*_vac_〉 exhibits a linear increase as a function of fluence, as reported in Figure 6. This trend can be fitted by the following relation:

(5) $$\langle N_{\text{vac}}\rangle =\langle N_{\text{vac},0}\rangle +\nu \sigma N_{\text{gr}}\Phi$$

where 〈*N*_vac,0_〉 is the extrapolation of the average vacancy density at *Φ *= 0, *σ *is the cross section for direct *C*-*C *collisions, *N*_gr _is the *C *density in a graphene sheet (*N*_gr _= 4 × 10^15 ^cm^-2^), and ν is the vacancy generation efficiency. By linear fitting the data in Figure 6, 〈*N*_vac,0_〉 = (1.59 ± 0.04) × 10^10 ^cm^-2 ^and *νσN*_gr _= (8.55 ± 0.06) × 10^-4 ^are obtained. For the calculated values of the *C*-*C *scattering cross section *σ*, ranging from 2 × 10^-17 ^to 7 × 10^-17 ^cm^2^, a very low vacancy generation efficiency (ranging approximately from 0.3 to 1.1%) is obtained for graphene irradiation with 500 keV C^+ ^ions. It might be associated to a dynamical annealing, e.g. vacancy-interstitial recombination, during irradiation.

## Conclusions

In summary, the authors propose an innovative method based on local capacitance measurements to probe the local changes in graphene electron mean free path, due to the presence of charged impurities or point defects, e.g., vacancies. Irradiation with 500 keV C^+ ^ions at fluences ranging from 1 × 10^13 ^to 1 × 10^14 ^cm^-2 ^was used to introduce defects in SLG deposited on a SiO_2_/n^+^Si substrate. The local charged impurity and vacancy density distributions were determined for the different irradiation fluences, and a low efficiency of vacancy generation (approximately from 0.3 to 1.1%) was demonstrated.

## Abbreviations

2D: two-dimensional; HOPG: highly oriented pyrolytic graphite; SCM: scanning capacitance microscopy; SCS: scanning capacitance spectroscopy; SLG: single layers of graphene.

## Competing interests

The authors declare that they have no competing interests.

## Authors' contributions

FG and VR conceived the study. FG coordinated the experiment, participated to the analysis of the data and wrote the article. SS carried out the sample preparation, the measurements and participated to the analysis of the data. ER worked on the evaluation of ion-graphene interaction cross sections. All the authors read and approved the manuscript.
